# A Case in Which a Sharp-pointed Body Was Swallowed by a Child, Passing the Bowels without Injury

**Published:** 1845-03

**Authors:** B. W. Avent

**Affiliations:** Murfreesboro’, Tenn.


Art. II.—*A Case in which a sharp-pointed body was swallowed by a child, passing the bowels without injury.* By B. W. Avent, of Murfreesboro', Tenn.

On Thursday evening, 8th July, I was requested to visit a little girl, four years old, who, whilst engaged at play, had accidentally swallowed a sharp-pointed instrument, about two and a half inches long. This instrument was originally the handle of a long-bladed knife, the jaws of which had been filed off about its centre, leaving the back-spring, which had been ground very sharp at its point.

I saw the patient an hour after the accident had occurred, and as might have been expected under such circumstances, found the family in great alarm, and in the act of preparing an emetic, with a view to cause the stomach to eject this foreign body.

The little girl was suffering no pain at all, and on examination I was satisfied that the instrument had passed the cardiac orifice without producing any injury in its passage. Aware that the point of this instrument was sufficiently sharp to penetrate the stomach, should it come in contact with it during any contractile action of that organ, I at once explained to the parents the great danger of medical interference, and advised that the unassisted efforts of nature should be relied upon for relief, at least until some unpleasant symptoms should arise. With this advice, I left the patient about as comfortable as if nothing unusual had happened.

On the following morning she complained of some pain in the epigastrium, but it was not of sufficient violence to excite much alarm. She took her breakfast as usual, and was permitted to engage in her accustomed amusements. After the morning, the pain in the stomach subsided. Sometime during the day her bowels were evacuated, without presenting any unusual appearance in the fæces.

On the next (Saturday) morning, she was still well, had no fever, or visceral excitement whatever, and had complained of no pain since the morning of the previous day.—Through this day she continued to be playful, and suffered no inconvenience. The bowels were once moved without medicines.

Sunday morning.—Patient still free from all excitement. At 9 o'clock this morning, just 64 hours after the occurrence of the accident, the instrument was discharged from the bowels, enveloped in fæces, without any pain, or inconvenience whatever. But little, if any, visible impression had been produced upon it during its passage through the bowels.

In the management of this case I applied no medical treatment, though often solicited to do so. I advised that the little patient should be permitted to engage in her customary amusements, and to take her ordinary diet, hoping by this course to keep her system, as nearly as possible, in a normal condition, the natural action of the alimentary canal undisturbed, and that thus, as happened, the 'unwelcome visitor' might be expelled.

Medical interference, in this case, would have consisted either in vomiting, with a view to the ejection of the contents of the stomach, or in the use of purgatives, more speedily to evacuate the contents of the bowels. In either plan of treatment there would have been great danger to the patient. The contraction of the stomach, necessary in vomiting, would undoubtedly have endangered the wounding its coats, by coming in contact with the sharp point of the instrument, at every effort of that character, to say nothing of the great improbability of effecting the object desired; whilst, on the other hand, cathartics would not only have produced irritation of the bowels, but by carrying off too hastily their fæcal contents, might have left the foreign body behind, in a condition to wound them at every peristaltic motion.

Two circumstances existed in this case which favored the safety of the patient. First, the instrument was swallowed with the handle or blunt end downwards, which prevented its wounding the parts in its passage; and, secondly, its length prevented its taking a transverse position; either of which circumstances might have greatly endangered, if not destroyed, the life of the patient.

Murfreesboro', January, 1845.

